# Editorial: Sexual dimorphism and steroid hormone crosstalk, volume II

**DOI:** 10.3389/fendo.2023.1346433

**Published:** 2024-01-03

**Authors:** Leandro Fernández-Pérez

**Affiliations:** Farmacología Molecular y Traslacional, Instituto Universitario de Investigaciones Biomédicas y Sanitarias (IUIBS), Universidad de Las Palmas de Gran Canaria, Las Palmas de Gran Canaria, Spain

**Keywords:** steroids, dimorphism, testosterone, GH, liver, transcriptome

Men and women diverge in their vulnerability to disease risk and progression as well as in the way they respond to drug metabolism and therapy. Many examples of sexual dimorphism exist in human and the prevalence of different cancers, autoimmune disorders, infectious diseases, cardiovascular or neurological diseases differs between men and women. Clinically relevant is the colon cancer therapy where men and women tend to respond differently to chemotherapy. Despite these evidences, most basic and clinical research has been carried out on male subjects because researchers like “to remove the influence of female hormones as a variable”, which has added to scientific literature a male-biased information. Since sexual dimorphism has impacted the preclinical scientific process itself and how clinical medicine should be carried out, it is highly recommended to include both males and females in both preclinical and clinical studies. If not, a drug that on a population level (i.e., without a stratified analysis) might not show a significant effect but in a stratified analysis might prove to be more effective in men than in women. Historically, a lot of the work has pointed to how steroid hormones regulate differential gene expression between male and females. Most findings have shown that steroid hormones exert tissue actions through local nuclear receptors, and/or indirect effects, by interactions with pituitary hormones. However, the molecular and signaling mechanisms that turn the genes on and off still deserve further research both in animal and human. Five of the manuscripts discussed below, although attached to this Research Topic, have appeared in a different sections of Frontiers in Endocrinology. In this Research Topic, a transcriptomic and lipid profiling analysis revealed a functional interplay between testosterone and Growth Hormone in hypothyroid rat liver (Fernández-Pérez et al.). This study reinforces the hypothesis that the liver is a primary organ of interactions between testosterone and GH. Testosterone positively cooperated with the male pattern of GH administration to support a male phenotype and liver transcriptome. This comprehensive analysis of gene expression and lipid profiling in rat liver revealed a functional interplay between testosterone and the male pattern of GH administration which significantly differed from the crosstalk between estradiol and GH. The effects of steroid hormones on lipid metabolism in sexual dimorphism were also studied in human (Liang et al.). Interestingly, the relationship among estrogen deficiency and sarcopenia development was systematically investigated in a rat model of ovariectomy-induced sarcopenia (Shu et al.). Notably, this bioinformatic analysis identified an integrated model combining obesity, osteoporosis and sarcopenia. This analysis confirmed the importance of estrogen in the maintenance of skeletal muscle function and homeostasis, and provided potential targets for further study on steroid hormone-related sarcopenia. Notably, these authors identified steroid hormones (DHEAS, progesterone, and androstenedione) with beneficial effects on lipid metabolism in both sexes; however, the specific lipid profiles affected by steroid hormones differed between the sexes. A link between somatotropic-liver axis, sex steroids and lipid metabolism was also studied in Igf1R deleted mice (Pérez-Matute et al.). The authors demonstrated that Igf1R was involved in metabolic homeostasis, with effects modulated by diet-induced obesity and aging in a sex dependent manner. These findings could contribute to understand metabolic alterations observed in patients with IGF1R gene deletions. Several phenotype and serum biomarkers linked to the biological effects of sex hormones in human, which are clinically relevant, were also studied in this Research Topic. First, the effect of endogenous testosterone on females was studied in PCOS, a female masculinization disease (Yan et al.), where the “digit ratio” could be used as phenotype biomarker of testosterone activities. Second, precocious puberty was also correlated with a large BMI (Liu et al.). In this population study, the authors concluded that precocious puberty was correlated with a large BMI and boys had a higher threshold of BMI for puberty development than girls. Third, bioinformatic analysis identified a immunological profile of Turner syndrome with different X chromosome origins (Qi et al.). This study indicated that signaling pathways involved in cancer and immune response were suppressed, findings that still need further studies for functional validation of these genes. Finally, a partial androgen insensitivity might be the most important cause of 46,XY differences of sex development (DSD) in children, whereas congenital adrenal hyperplasia was the most common cause of 46,XX DSD (Abdelghaffar et al.). In addition, this study described the importance of biomarkers such as serum anti-Müllerian hormone and inhibin B in detecting the presence of functioning testicular tissue. In summary, this Research Topic “*Sexual Dimorphism and Steroid Hormone Crosstalk*” explored the influence of sex hormones on rodent and human gene networks and how they could contribute to sexual dimorphism. From a clinical point of view, finding differences between sexes that control drug metabolism and drug response, or the vulnerability to disease risk and progression will help to perform a more precise personalized Medicine. This is a scientific and social challenge because the combination of all sex specific genetic, epigenetic, and hormonal influences of biological sex produces unique *in vivo* environments for female and male cells. Building computational (i.e., artificial intelligence), statistical, and quantitative methods will highlight the most determinant genetic, epigenetic, and sex steroids drivers of sexual dimorphism in risk disease and response to therapy. A better ability to predict disease risk, disease evolution, and more efficient therapies for any individual patient will be improved by integrating in these models the sex of the patient.

## Author contributions

LF-P: Conceptualization, Investigation, Supervision, Validation, Writing – original draft, Funding acquisition, Writing – review & editing.

